# Prevention and Therapy of Type 2 Diabetes—What Is the Potential of Daily Water Intake and Its Mineral Nutrients?

**DOI:** 10.3390/nu9080914

**Published:** 2017-08-22

**Authors:** Johannes Naumann, Diana Biehler, Tania Lüty, Catharina Sadaghiani

**Affiliations:** Interdisciplinary Center for Treatment and Research in Balneology, Institute for Infection Prevention and Hospital Epidemiology, Medical Center–University of Freiburg, Faculty of Medicine, University of Freiburg, 79106 Freiburg, Germany; diana.biehler@uniklinik-freiburg.de (D.B.); tania.luety@uniklinik-freiburg.de (T.L.); catharina.sadaghiani@uniklinik-freiburg.de (C.S.)

**Keywords:** diabetes, water intake, mineral water, magnesium, bicarbonate, review, prevention

## Abstract

We aim to present an overview of the possible influence of drinking water in general and mineral water in particular in improving glycemic parameters in persons with or without type 2 diabetes. We performed a literature search that produced 15 randomized controlled trials (RCTs) on this topic with mainly small sample sizes. We also discuss relevant observational and animal studies as well as the effects of important supplements in mineral water such as hydrogencarbonate and magnesium. There is low evidence for the positive effects of water or mineral water in improving glycemic parameters in diabetic and non-diabetic persons, and the results are heterogenous, making it difficult to reach an unequivocal conclusion. Meta-analyses of prospective cohort studies and other observational studies, studies with animal models and interventional studies using hydrogencarbonate and magnesium supplements suggest a probable positive effect of drinking water and mineral water in particular on glycemic parameters, supporting the positive results found in some of the RCTs, especially those substituting diet beverages or caloric beverages with water, or those using bicarbonate and magnesium-rich water. Regarding the high prevalence, the associated suffering and the resulting health expenditures of type 2 diabetes, it is imperative to conduct larger and more rigorous trials to answer the question whether drinking water or mineral water can improve glycemic parameters in diabetic and non-diabetic persons.

## 1. Introduction

The incidence of type 2 diabetes mellitus, pre-diabetes, impaired glucose tolerance and metabolic syndrome has increased considerably worldwide. The global prevalence of diabetes is estimated to be 8.5%, i.e., there are 422 million diabetics worldwide and 59.8 million diabetics in Europe (9.1%) alone [1,2]. 

Making lifestyle changes including a healthy diet and sufficient physical activity of 150 min a week, which should result in a weight loss of at least 7% [3], is the most effective way to reduce the risk of developing diabetes by an estimated 34–43%. Furthermore, a diet and physical activity can reduce diabetes incidence, cardiovascular (hazard ratio (HR) 0.59, 95% confidence interval (CI) 0.36–0.96; *p* = 0.033) and all-cause mortality (HR 0.71, 95% CI 0.51–0.99; *p* = 0.049) [4]. 

A healthy diet should include beverages with low sugar and calorie content, but not diet (artificially sweetened) beverages, because habitual consumption of sugar sweetened drinks, sweetened-milk beverages [5] and diet beverages [6] is associated with higher type 2 diabetes risk.

People who don’t drink sugar sweetened beverages tend to have higher quality diets and do not compensate for sugar or energy deficits by consuming more sugary foods [7].

A nutrition survey examining the relationship between the quantity of water taken in and HbA1c in 1035 participants calculated that an increase of 240 mL in daily water intake reduces the risk of elevated HbAc1 >5.5% by 22% in men but not in women [8]. 

A review of 134 randomized controlled trials (RCTs), where water consumption was increased right before or during a meal, showed heterogenic effects on energy intake, energy expenditure, fat oxidation and weight change [9].

Moreover, mineral nutrients such as bicarbonate [10] and magnesium [11], in particular, are thought to affect glucose metabolism. An increased dietary acid load is associated with the development of insulin resistance [12], while a pre-existing diabetes favors acidosis, which, in turn, leads to increased insulin resistance [13].

Drinking recommendations are less common and detailed than food recommendations [3,14], and there is little scientific evidence, and, to our knowledge, no review of RCTs on drinking water and its influence on glycemic parameters, respectively type 2 diabetes.

This review aims to explore to what extent drinking water with low or high mineralization for at least four weeks influences glycemic parameters in healthy or type 2 diabetes participants in daily life situations as compared with other drinks. The results of the RCTs found in the literature search will be discussed alongside observational studies, animal studies, pathophysiological aspects as well as results from interventional studies using mineral supplements. 

## 2. Methods 

### 2.1. Overview

Two reviewers (J.N., D.B.) identified RCTs as well as crossover RCTs reporting long-term (≥4 weeks treatment) effects of drinking water on glycemic parameters.

### 2.2. Study Search

The Medline database was searched via PubMed for fully published peer-reviewed articles from the time of database inception to 8 August 2017. The search strategy is outlined in Table 1. A systematic literature search was not performed since the focus was to evaluate the potential, not to estimate unbiased effects of drinking water and its mineral nutrients in prevention and therapy of type 2 diabetes.

### 2.3. Study Inclusion and Exclusion Criteria

The criteria were as follows:

(1) Types of study: RCTs were the focus of this review to restrict potential sources of error and facilitate inference about possible causal mechanisms. They were included regardless of quality measures, such as double-blind design, complete protocol adherence, patient attrition, similarity of treatment and control groups at baseline and intention-to-treat analysis or sample size, target population or unit of analysis (e.g., individual or group-level data). They were only eligible if they were published as full paper articles. No language restrictions were made.
(2) Types of participants: individuals of all ages (whether healthy or with or without type 2 diabetes, at risk of type 2 diabetes or other diseases, as having cardiovascular risk factors (CVRF), being overweight or obese or not, with hyperlipemia or not; excluded: type 1 diabetes).
(3) Types of intervention: plain or drinking water, served at any temperature, whether tap or bottled water, high or low mineralized, carbonated or uncarbonated water.
(4) Types of comparisons: interventions or control groups with drinking water tested alone, as a single intervention or as main part of a multi-component intervention for at least four weeks.
(5) Types of outcome: at least one parameter for glycemic control as shown in Table 2 independent whether it was a primary or secondary outcome of the study.

Studies excluded were those that measured only short-term effects of drinking water on glycemic parameters because short-term effects may not well reflect the influence on incidence and outcome of type 2 diabetes. Studies investigating severe diseases or special situations, such as, for example, acute gastroenteritis or extreme sport or climatic situations were also excluded. 

### 2.4. Data Extraction and Risk of Bias Evaluation

The authors (J.N., D.B.) of the review present here independently extracted relevant study information (for example, population, characteristics of the intervention and control, outcome measures, results) using predefined data sheets, including the Cochrane risk-of-bias indicators (J.N., C.S.) [15]. We report the items which are of high or unclear risk of bias regarding the key domains—selection, performance, detection, attrition and reporting bias. If necessary, existing inconsistencies were solved by discussion, and consensus achieved. 

## 3. Results

### 3.1. Literature Search

**Figure 1:**
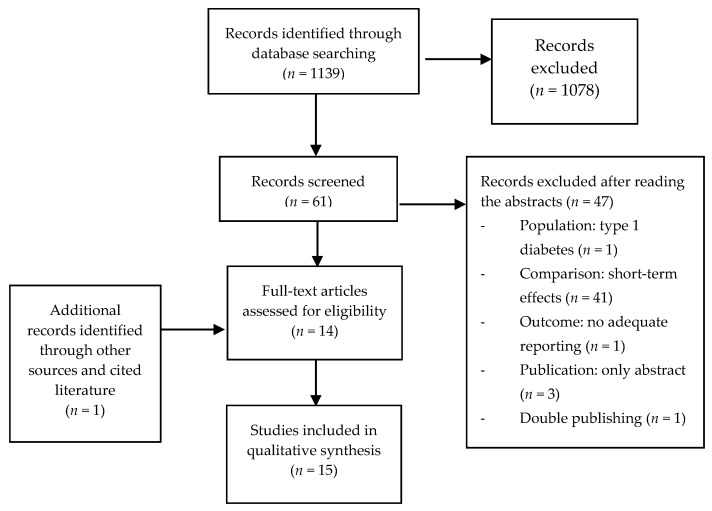
Flow-chart of the literature search.

Our literature search yielded 1139 articles, 1078 of which were excluded by two independent reviewers (J.N., D.B.) based on their titles (other intervention or outcomes or study type), thus leaving 61 trials (Figure 1). 

After reading the abstracts, and, if necessary, the whole article, another 47 trials were excluded:

(a) Population: 1 (type 1 diabetes)
(b) Comparison: 41 trials only investigated short-term effects (<4-weeks treatment)
(c) Outcome: 1 (glycemic parameter measured, but not reported)
(d) Publication: 3 only abstracts (1 Russian, 1 Japanese, 1 Italian)
(e) Double publishing: 1

After reviewing references, one additional RCT [16] was included, which is not indexed in Medline or web of science. Hence, 15 studies were reviewed.

### 3.2. Description of Included Studies

The characteristics of the included studies are detailed in the following Table 3, Table 4 and Table 5. 

The studies were separated according to mineralization of the water and the control group:

Table 3: RCTs comparing water with other drinks or no drink (eight studies); [17,18,19,20,21,22,23,24].

Table 4: RCTs comparing low mineralized with bicarbonate-rich water (five studies); [25,26,27,28,29].

Table 5: RCTs comparing low mineralized with bicarbonate- and magnesium-rich water (two studies); [16,30].

Regarding the study populations investigated, we included four studies with type 2 diabetes [19,22,23,24], three with overweight or obese [17,18,21], three with moderately elevated cholesterol [26,27,29], one with at least one CVRF [20] and four with healthy participants [16,25,28,30].

Three studies investigated women [18,19,25], one of them postmenopausal women [25], two studies men [16,29] and ten studies both genders [17,20,21,22,23,24,26,27,28,30]. One study investigated elderly people (60–72 years) [28].

Of the included studies, eight compared water with other beverages (Table 3): diet beverages [17,18,19], artificially sweetened cola, sucrose sweetened cola and semiskim milk [21], blueberry juice or no water [20], red or white wine [23], red wine with or without alcohol [22], and chamomile tea [24]. 

A further seven studies compared different types of water, all low mineralized with bicarbonate-rich water (879–2946 mg/L), five of them with low [25,26,27,28,29] and two [16,17,30] with high concentrations of magnesium (102 and 291 mg/L) (Table 4 and Table 5); three studies compared uncarbonated water [16,28,30], two studies carbonated water [27,29] and two studies carbonated bicarbonate-rich water versus uncarbonated mineral water [25,26].

All 15 studies reported fasting blood glucose, 12 studies fasting insulin or C-peptide levels (not reported in [17,25,29]) of which seven calculated HOMA-IR; (Homeostatic Model Assessment for Insulin Resistance) [18,19,21,22,23,24,30]. Four of the studies additionally conducted glucose tolerance tests (GTT) [16,18,19,28], one reported serum fructosamin concentrations [16], and one plasma glycoalbumin concentration [30].

## 4. Discussion

### 4.1. Results of RCTs Comparing Water with Other Drinks or No Drink (Table 3)

In an RCT, 318 obese participants were advised to replace two caloric beverages (each 350–500 mL) a day with water or diet beverages for six months or to stay with the usual drinks. This resulted in a significant increase of water consumption of 1 L after three months and 0.8 L after six months and a significant decrease of fasting plasma glucose (FPG) after six months in the water group compared to the control group with no change of beverages, whereas replacement with diet beverages showed no significant difference [17].

Two trials [18,19] reported significant positive effects on body weight and glycemic parameters when replacing 250 mL of an diet beverage with water once a day in overweight and obese women participating in a weight loss program for 24 weeks. One trial investigated otherwise healthy women, the other trial women with well controlled type 2 diabetes. In both trials, the intake of calories and carbohydrates was significantly lower in the group drinking water instead of diet beverages, which explains at least partially the positive effect on the glycemic parameters. This result is supported by an observational study in the USA comprising 18,311 participants, which showed that a higher plain water intake was accompanied by a decrease in total intake of calories, sugary beverages, total fat, saturated fats, sugar, sodium and cholesterol [32].

Conversely, another RCT [21] comparing consumption of 1 L of low mineralized uncarbonated water with an diet beverage (aspartame sweetened Cola) for six months showed no significant difference between the groups in FPG, Insulin or HOMA-IR; rather, a positive trend for diet cola. This is surprising because several meta-analyses of observational studies [6,33] and a survey of 75 countries [34] found a positive correlation between diet beverages and type 2 diabetes, sometimes even higher than for sucrose sweetened beverages. Furthermore, the amount of water and duration of the intervention were sufficient, as a nine-year follow-up of 3615 participants showed a significant lower risk of hyperglycaemia (20–30%) in participants drinking more than 0.5 L of water daily compared with drinking less [35], and an increase of 240 mL in water intake revealed a significant decline in the type 2 diabetes risk score in a cross-sectional study [36]. The authors of the study discuss the small sample size as a possible factor for this finding.

In the RCT discussed above [21], water was also compared to sucrose sweetened cola and semi-skimmed milk, both of which showed no significant difference between the groups for FPG, Insulin or HOMA-IR. The lack of a significant difference between water and cola is also surprising because two meta-analyses of observational studies show a higher incidence of type 2 diabetes with consumption of sucrose sweetened beverages [37,38], whereas the lack of a significant difference between water and milk is in accordance with the literature. A meta-analysis of observational studies on total low-fat and high-fat milk intake was not associated with an increased risk for type 2 diabetes, and yogurt intake of 80 g/day was even nonlinearly inversely related with a 14% lower risk [39].

A further three studies showed no significant difference in the glycemic parameters. 

One study [20] compared consumption of 1 L tap water per day for four weeks with drinking 1 L blueberry juice or no drink at all. Total calorie intake was not reported, thus it is not clear how the additional calorie intake from the juice was compensated. In a recent meta-analysis of observational studies, fruit juice consumption resulted in a 5% increase of incidence of type 2 diabetes [6]; however, a correlation between fruit juice consumption and the incidence of type 2 diabetes was not found in a European meta-analysis [37], and an observational study from Costa Rica substituting sucrose sweetened or diet beverages with fruit juice showed a trend towards better glycemic parameters and a lower incidence of metabolic syndrome than substituting it with water [40].

Another study [22] found no significant effects when comparing the effects of drinking 230 mL (women) or 300 mL (men) red alcoholic or dealcoholized wine or water once a day in the evening over four weeks [22] on CVRF, including glycemic parameters. The amount of water may have been too small for positive effects on glycemic parameters and total calorie intake was not reported. 

The third study [23] investigated the cardiovascular effects of 150 mL red or white wine once a day compared to water over two years, and found no significant difference for red wine for all glycemic parameters, and for white wine even a significant decrease of FPG and HOMA-IR compared to water. The diet was comparable (Mediterranean Diet recommended) and, as the authors state in their discussion, the better result for the white wine may be a chance finding. The amount of water consumed in these studies may be too low for any effects on glycemic parameters.

The results of the last two studies are in accordance with a recent meta-analysis with moderate amounts of alcohol showing benefits on glycemic parameters [41].

In a further study (published twice) [24,31], drinking three times 150 mL of chamomile tea per day showed a significant decrease for HbA1c, serum insulin and insulin sensitivity compared to 150 mL hot water. As chamomile tea contains the same amount of water, this study does investigate the effect of the water itself, rather that of chamomile, particularly as the additional amount of hot water was probably compensated through reduction of other drinks. The antidiabetic effect of chamomile is supported by several studies in diabetic rats, as the authors state in their discussion. 

### 4.2. Results of RCTs Comparing Low Mineralized Water with Bicarbonate-Rich Water with Low Magnesium (Table 4)

One study with 18 healthy postmenopausal women showed a significant decrease of a glycemic parameter, FPG, for use of 1 L of carbonated bicarbonate-rich water for two months compared to 1 L of an uncarbonated low mineralized water [25].

The other four studies found no significant difference between the use of bicarbonate-rich water compared to low mineralized water for the investigated glycemic parameters.

One of these studies, with a sample size of 18 healthy adults, found a trend (*p* = 0.056) for FPG after eight weeks of 1 L of carbonated bicarbonate-rich water compared to a carbonated low mineralized water [27].

The largest study with 64 healthy adults showed a significant decrease of FPG compared to baseline for carbonated bicarbonate-rich and uncarbonated low mineralized water, with no significant difference between the groups [26]. The study showed a significant increase in total fluid intake with a significant decrease of consumption of soft drinks and fruit juice and a trend toward less beer, showing that the water was replacing other drinks. Results also showed a significant lower intake of calories and carbohydrates for both water groups. This result reflects partially a regression to the mean and partially a positive effect of substituting other beverages and food with water.

One study with 16 healthy elder adults (60–72 years) on a salt restricted diet, compared two different bicarbonate-rich waters with low mineralized water, one with a higher sodium than bicarbonate content. This trial showed no difference in FPG, and the decrease in insulin in favor of the sodium rich water (decrease of insulin −0.5 vs. −0.9 mU/L) was too small to reach significance [28]. In so far as this result has any significance, it is supported by a meta-analysis of 19 crossover RCTs (*n* = 494), which found higher fasting insulin concentrations with sodium reduction [42].

The other crossover RCT, in which 12 healthy men consumed 1.25 L water with the highest content of bicarbonate of the searched studies (4168 mg/L) for eight weeks, also found only a small non-significant difference of 3 mg/dL for FPG in favor of the bicarbonate-rich water [29]. 

None of the five studies had a sample size larger than 64 participants and four studies fewer than 18 (range 12–18) and therefore did not have sufficient power to detect small or even moderate effects.

### 4.3. Results of RCT Comparing Low Mineralized Water with Bicarbonate-Rich Waters with High Magnesium (Table 5)

The first study compared consumption of 1.4 L of a bicarbonate-rich water with a content of 102 mg/L magnesium for four weeks with an uncarbonated low mineralized water [16]. In spite of the small sample size, the trial found a small but significant difference for serum fructosamin for the bicarbonate- and magnesium-rich water compared to the low mineralized water. Furthermore, the glucose tolerance test showed a significantly better result in the bicarbonate-rich water group, but there was no significant difference for the short-term glycemic parameters FPG and fasting insulin.

The other study [30] investigated a bicarbonate-rich water with an even higher concentration of magnesium (291 mg/L) and also showed a significant better result in the long-term glycemic parameter glycoalbumin compared to low mineralized water and no significance for the short-term glycemic parameters FPG, fasting insulin and the calculated HOMA-IR.

As shown below, the positive results may well be due to the high magnesium content. However, it is also apparent that the positive significant results were found only in longer-term glycemic parameters, indicating that the effects from water, especially bicarbonate-rich water, may be too small to detect them in the short-term parameters FPG, fasting insulin and HOMA-IR, but large enough to result in a moderate effect in longer terms.

The two studies [16,30] had a small sample size comprising 23 and 19 healthy participants, respectively. They therefore did not have sufficient power to detect small or even moderate effects. Both studies found significant positive results for long-term glycemic parameters (fructosamin and glycoalbumin).

#### 4.3.1. Effect of Bicarbonate as a Supplement

All studies comparing the effect of tap water and mineral water used bicarbonate-rich mineral water, since a high dietary acid load is associated with increased insulin resistance as well as with disturbed glucose metabolism. The following section highlights some studies investigating the effect of bicarbonate on glycemic parameters.

A recent RCT [43] in diabetic patients with chronic kidney disease showed that, compared with a placebo, supplementation with bicarbonate targeting a serum concentration of 24–28 mmol/L significantly improved multiple parameters of glucose metabolism. For example, the serum glucose concentration decreased from 127 mg/dL to 110 mg/dL, the proportion of glycated haemoglobin declined from 7.7% to 6.7%, insulin resistance (HOMA-IR) decreased from 6.1 to 7.0, and serum insulin concentration decreased from 13.4 mcIU to 19.9 mcIU. Insulin resistance declined up to a bicarbonate concentration of 28 mmol/L; however, it increased when 28 mmol/L of bicarbonate was exceeded. Thus, the improvement in insulin resistance was based on a correction of acidosis rather than on the serum bicarbonate concentration itself. Mild acidosis is accompanied by increased insulin resistance even in healthy subjects. 

A cross-sectional study [44] investigated the relationship between serum hydrogen carbonate concentration and insulin resistance in 1496 healthy adults. Participants with lower serum hydrogen bicarbonate concentration showed both significantly increased insulin resistance and a significantly elevated serum insulin concentration. This effect was even more distinct in participants with higher BMI. Similarly, a recent study [12] revealed a relationship between an increased lactate concentration, which is an indicator for mild metabolic acidosis, and insulin resistance in 104 sedentary adults. 

The relevance of serum hydrogen carbonate in prevention was reported in a prospective study in 630 women (Ø = 56 years) and a 10-year follow up. Women with lower serum hydrogen carbonate concentrations had a significant higher risk of developing type 2 diabetes than women with higher serum hydrogen carbonate concentrations [10].

#### 4.3.2. Effect of Mineral Rich Water in an Animal Model

It is known that rats receiving 10% fructose in their drinking water develop symptoms of metabolic syndrome and that the plasma glucose level increases considerably. 

If fructose is administered in mineral water rather than tap water, the basic parameters of glucose metabolism, e.g., glucose, insulin and triglyceride concentrations, do not deteriorate at all or at least less [45]. Similarly, rats receiving fructose in tap water showed a strong increase in aldosterone level, whereas rats receiving fructose in mineral water had the same level as the control rats that did not receive any fructose. A review from 2016 [46] describes the relationship between elevated aldosterone level and both metabolic syndrome and type 2 diabetes.

Sirtuin 1 (SIRT1) and peroxisome proliferator-activated receptor-gamma coactivator (PGC)1-α ) are proteins that counteract metabolic syndrome and aberrant glucose metabolism. SIRT1 positively influences multiple steps in glucose metabolism in the liver, the pancreas, muscles, and adipose tissue. De-acetylated PGC1-α is the main regulator of the SIRT1 pathway [47,48]. The expression of both proteins was significantly higher in liver tissue of rats receiving mineral water compared to rats receiving tap water [49]. In addition, the amount of phosphorylated insulin receptor substrate (IRS), the active form of the substrate that forwards the insulin signal, was elevated in liver tissues of rats receiving mineral water compared with rats receiving tap water [50]. This indicates increased insulin sensitivity. However, both groups of rats taking in fructose had a lower level of phosphorylated IRS compared with control rats. This might be due to the fact that the serum magnesium concentration in rats receiving fructose significantly declined (1.71 mmol/L and 1.55 mmol/L, respectively) [45].

Magnesium ions are essential for the auto-phosphorylation of the insulin receptor, as two magnesium ions bind the tyrosine kinase domain. Disruption of phosphorylation is considered the main mechanism leading to insulin resistance through lack of magnesium ions [51].

In summary, these animal experiments suggest that intake of mineral water in patients with either metabolic syndrome, pre-diabetes, or type 2 diabetes might have a positive influence.

Besides bicarbonate, magnesium seems to be important. Both RCTs studying effects of mineral water rich in magnesium determined long-term blood glucose concentrations. Hence, the positive effect observed in these studies cannot be traced back exclusively to magnesium. Still, the currently available data indicate a positive influence of magnesium on the glucose metabolism and one might assume that magnesium absorbed from mineral waters also positively influences glucose metabolism.

### 4.4. Clinical Trials Considering Magnesium

Results from studies investigating mineral waters containing relevant concentrations of magnesium are consistent with the results from studies investigating magnesium supplementation or intake of dietary magnesium. The latter demonstrated that in subjects suffering from magnesium deficiency, pre-diabetes, or insulin resistance, additional magnesium intake may ameliorate their glucose metabolism. Indeed, in 51% of the subjects suffering from magnesium deficiency and pre-diabetes, magnesium supplementation of 382 mg/day positively influenced glucose metabolism compared to 7% of the subjects in a placebo group [52]. In adults with low insulin sensitivity, magnesium supplementation of 365 mg/day caused the serum glucose level to drop significantly and increased insulin sensitivity [53]. 

The most recent systematic review from 2016 including a meta-analysis of 21 RCTs in 1362 participants (684 individuals in Mg^2+^ group and 678 individuals in control group) compared the influence of magnesium supplementation on insulin sensitivity and glucose metabolism in diabetic individuals with the influence in non-diabetic individuals [11]. This review demonstrated significant amelioration of insulin resistance in both diabetic and non-diabetic individuals. A significant reduction of the serum glucose concentration was observed in a subset of studies including only those with supplementation for at least four months and only considering participants with magnesium deficiency. 

#### 4.4.1. Magnesium Blood Concentration

In 2012 [54], a case control study in 200 obese individuals revealed that the 50 diabetics had a lower serum magnesium concentration than the 150 participating non-diabetics. Forty-eight per cent of the diabetics had magnesium deficiency compared with only 15% of the non-diabetics. Furthermore, a lower magnesium concentration was associated with a higher plasma glucose level, higher insulin resistance and a higher HbAc1 concentration.

A prospective study explored this relationship in 8555 participants with a follow-up period of 5.7 years [55]. A reduction in the serum magnesium concentration of 0.1 mmol was associated with an 18% higher risk of developing diabetes (HR = 1.18; 95% CI = 1.04–1.33) and a 12% higher risk for pre-diabetes (HR = 1.12; 95% CI = 1.01–1.25). In participants deficient in magnesium, the diabetes risk increased by 79% (HR = 1.79; 95% CI = 1.16–2.77) and the pre-diabetes risk increased by 44% (HR = 1.44; 95% CI = 0.91–2.27), however, the latter finding was not significant. 

#### 4.4.2. Dietary Magnesium

In 2011, a review with meta-analysis of 13 prospective studies in a total of 536,318 participants and 24,516 diabetes cases [56] showed a significantly reduced risk for type 2 diabetes: Increasing the daily magnesium intake by 100 mg was accompanied by a decrease in diabetes risk by 14%. 

The most recent meta-analysis of 25 prospective studies comprising 637,922 participants revealed the following dose-effect-correlation: An increase of daily magnesium intake by 100 mg was associated with an 8–13% lower diabetes risk [57].

Another meta-analysis including 6 cross-sectional studies in a total of 24,473 participants investigated the correlation between magnesium intake and metabolic syndrome [58]. This study revealed that daily intake of 100 mg magnesium was associated with a risk reduction by 17%.

#### 4.4.3. Relevance of Magnesium for Glucose Metabolism 

A review by Gommers et al. [51] summarizes the multiple functions of magnesium on a molecular level. Another narrative review by Mooren [59] sums up the molecular mechanisms and links them to data from clinical trials. 

The binding of insulin to its receptor triggers a signaling cascade leading to import of glucose into the cell (muscle), to glycogen synthesis (liver) and lipid synthesis (adipose tissue). If this signaling cascade is impaired, more insulin is released into the bloodstream. When the increased amount of insulin is in the end also unable to trigger the signaling cascade sufficiently, blood sugar levels eventually rise.

Magnesium plays a direct role in the signaling cascade as a co-factor of the insulin receptor. Magnesium binds to the intracellular domain of the insulin receptor, thereby allowing or enhancing transmission of the insulin signal induced by the extracellular binding of insulin to the receptor. Hence, magnesium increases insulin sensitivity.

It is known from studies in mice, that oral magnesium supplementation increases the number of glucose transporters in the cell membrane of muscle cells; glucose can therefore be imported faster and more effectively [60]. 

An inflammatory environment also contributes to development of insulin resistance and is one of the main reasons for an obesity-related elevated diabetes risk. Thus, magnesium as an anti-inflammatory ion can counteract insulin resistance [61]. 

To prevent diabetes, it is essential to preserve the function of β-cells [62]. A prospective study comprising 228 patients with disturbed glucose tolerance showed that the diabetes risk was smallest in participants with functioning β-cells at the beginning of the study [63]. The function of the β-cells was the strongest predictor for diabetes. In diabetic and non-diabetic participants, magnesium administration in the form of MgCl_2_ increased the function of β-cells [64].

Insulin also influences kidney cells to increase re-absorption of magnesium. After binding of insulin to its receptor, TRPM6 is recruited to the plasma membrane; consequently, magnesium re-absorption is enhanced [65]. Therefore, insulin resistance leads to increased magnesium excretion starting a vicious cycle. 

Most of the glucose ingested from food is absorbed into muscles. Deteriorated insulin resistance inhibits absorption into the muscles, whereby the reason for deterioration may either be magnesium deficiency or acidosis, in which bicarbonate might be an issue. 

### 4.5. Summary

In summary, the evidence for positive effects of water or mineral water in improving glycemic parameters in diabetic and non-diabetic persons is low and the results are heterogenous with no clear result. Positive results were mainly seen in studies where water replaced caloric or diet beverages, and where bicarbonate and magnesium-rich mineral water and long-term glycemic parameters were used.

Meta-analyses of prospective cohort studies and other observational studies, studies with animal models and interventional studies with supplements of hydrogencarbonate and magnesium suggest a probable positive effect of drinking water and especially mineral water on glycemic parameters, supporting the positive results found in some of the RCTs, especially those substituting diet beverages or caloric beverages with water and those using bicarbonate and magnesium-rich water.

## 5. Limitations and Generalizability

The main limitation of this review is the small number of RCTs and even more the small sample sizes in most of the RCTs that give the risk of overestimating the effects. The lack of blinding in most of the RCTs gives a relative high risk of bias compared to an otherwise relative low risk of bias in most of the studies.

Most of the RCTs did not measure long-term glycemic parameters and may therefore have missed positive effects as shown in the two RCTs using fructosamin and glycoalbumin [16,30].

The interventions lasted mainly four to eight weeks, only one study lasted six months and another two years. This may have been too short, as shown in the “Da Qing Diabetes Prevention Study” with 23-year follow-up, investigating diet and physical activity, which reported that the difference in the incidence of type 2 diabetes appeared only after two years [4].

Some of the interventions used relatively little amounts of water, especially when wine was the beverage being compared, possibly leading to negative results.

In this review, the generalizability is relatively high, as we only excluded severe diseases and special circumstances as extreme physical activity, extreme climate or acute or severe diseases. In addition, the population investigated in the studies covers nearly all ages; however, there are no studies with children or adolescents.

## 6. Conclusions

Water is an ideal calorie-free and sugar-free beverage, which may be suitable to reduce calorie and sugar intake, and if high mineralized water is also used to substitute those minerals that may be relevant for bicarbonate and magnesium.

Further research should focus on long-term glycemic parameters, and larger and more rigorous studies are needed to answer the question of whether drinking water or mineral water can improve glycemic parameters in diabetic and non-diabetic persons.

Regarding the high prevalence, the associated suffering and the resulting health expenditures of type 2 diabetes, it is imperative to conduct these RCTs.

## Figures and Tables

**Table 1:** Search strategy.

Search No.	Search Terms	Results	Search Fields
1	water	819,017	All Fields
2	glucose or glycemic or glycaemic or cholesterol	733,923	All Fields
3	drink* or consumption or beverage*	188,224	All Fields
4	study or trial* or review	10,225,821	All Fields
5	humans or women or men	16,761,909	All Fields
6	((((water) AND ((glucose OR glycemic OR glycaemic OR cholesterol))) AND ((drink* OR consumption OR beverage*))) AND ((study OR trial* OR review))) AND (humans OR women OR men)	1139	All Fields

**Table 2:** Parameters for glycemic control.

Parameters (with Normal Values) for Glycemic Control Reported in the Studies
**Fasting plasma glucose** (FPG < 110 mg/dL)
**Glucose tolerance test** (level 2 h after glucose loading < 140 mg/dL)
**Fasting insulin** (6–25 mU/L)
**HOMA-IR** (<2) (Homeostatic Model Assessment for Insulin Resistance)
(Product of fasting insulin (µU/mL) × fasting glucose (mg/dL))/405)
**Glycated hemoglobin, HbA1c (<6%)**
(Measure for blood glucose levels during the last 1 to 3 months)
**Glycoalbumin (11–16%)**
(Measure for blood glucose levels in the last 2 to 4 weeks)
**Fructosamin (200–290 µmol/L)**
(Measure for blood glucose levels in the last 1 to 3 weeks)

**Table 3:** Randomized controlled trials (RCTs) comparing water with other drinks or no drink.

Author/y	Study Type	Population	Intervention	Comparison	Outcome	Results	Risk of Bias
Tate et al., 2012 [17]	RCT parallel 3 groups	318 obese, BMI 36.3 kg/m^2^, 84% female, medium age 42 y, USA	Advice to replace more than 2 servings (each 350–500 mL) of caloric beverages per day with water for six months	CG1: Advice to replace more than 2 servings of caloric beverages per day with diet beverages CG2: no advice to change beverages, but general nutritional weight loss advices	FPG (SO) Weight loss (PO)	Water group had a sign. Increase of water consumption of 1 L after 3 months and 0.8 L after 6 months and a sign. Decrease of FPG compared to control group with no change of beverages after 3 and 6 months.	ITT, drop-outs 33/318 after 3 months and 46/318 after 6 months, no blinding
Madjd et al., 2015 [18]	RCT parallel 2 groups single-blind	62 healthy overweight and obese women, BMI 27–40 kg/m^2^, 18–50 y, non-smokers, participating in a weight loss program, Iran	250 mL tap water (not specified) per day after lunch for 24 weeks	250 mL diet beverage	FPG Fasting insulin HOMA-IR GTT Body weight (all PO)	Water group sign. more weight loss (8.8 kg vs. 7.6 kg *p* = 0.015) lower FPG (2.8 mU/L vs. 1.7 mU/L *p* < 0.001) better insulin sensitivity (HOMA-IR 1 vs. 0.8 *p* < 0.001) better GTT (1 mmol/L vs. 0.7 mmol/L, *p* < 0.001) compared to control group after 24 weeks.	No ITT, 9/71 drop-outs, measures of blinding not reported
Madjd et al., 2017 [19]	RCT parallel 2 groups single-blind	81 overweight and obese women, BMI 27–35 kg/m^2^ with type 2 diabetes HbA1c 6.5–7.2%, age 18–50 y, non-smokers, participating in a weight loss program with only metformin, Iran	250 mL tap water (not specified) per day after lunch for 24 weeks	250 mL diet beverage	FPG Fasting insulin HOMA-IR GTT Body weight (all PO)	Water group sign. more weight loss (−6.40 vs. −5.25 kg *p* = 0.006); lower FPG (−1.63 vs. −1.29 mmol/L *p* = 0.06) lower fasting insulin (−5.71 vs. −4.16 mU/L *p* < 0.011) better insulin sensitivity (HOMA-IR −3.2 vs. −2.48 *p* < 0.003) better GTT (−1.67 vs. −1.35 mmol/L *p* = 0.027) Compared to the control group after 24 weeks.	ITT, 16/81 drop-outs, measures of blinding not reported
Tonstad et al., 2006 [20]	RCT parallel 3 groups	67 men and 27 post-menopausal women (total *n* = 94) with at least 1 CVRF, Norway	1 L tap water (not specified) per day for 4 weeks	CG1: 1 L blueberry juice CG2: no change of habits	FPG Fasting insulin C-peptide (all SO) Blood viscosity (PO)	No sign. difference between groups after 4 weeks (exact data not presented)	No ITT, 5/99 drop-outs, no blinding,
Maersk et al., 2012 [21]	RCT parallel 2 groups	47 healthy overweight adults, 20–50 y, BMI 26–40 kg/m^2^, RR < 160/100, Denmark	1 L uncarbonated water per day for 6 months Aqua d’or HCO^3−^ 71 mg/L Cl^−^ 16.5 mg/L Na^+^ 14.9 mg/L Mg^2+^ 6 mg/L	1 L regular Cola (sucrose-sweetened) 1 L diet Cola, aspartame-sweetened 1 L semi-skim milk	FPG Fasting insulin HOMA-IR (all SO)	No sign. difference for FPG Insulin and HOMA-IR between groups	No ITT, 13/60 drop-outs, no blinding, sign. difference for sex at baseline (adjusted in analysis), randomization and allocation not reported
Mori et al., 2016 [22]	RCT crossover 3 groups	24 well controlled type 2 diabetes HbA1c < 8.5%, 19 men, 5 women, 40–70 y, regular alcohol intake women 20–30 g/day, men 30–40 g/day, Australia	Tap water (not specified) women 230 mL per day men 300 mL per day for 4 weeks	CG1: red wine women 230 mL/day (24 g alcohol/day) Men 300 mL/day (31 g alcohol/day), CG2: dealcoholized red wine women 230 mL/day Men 300 mL/day	FPG Fasting Insulin HOMA-IR (all SO) CVRF (PO)	No sign. difference for FPG Insulin and HOMA-IR between groups	No ITT, 4/28 drop-outs, no blinding
Gepner et al., 2015 [23]	RCT parallel 3 groups	224 alcohol-abstaining adults type 2 diabetes, HbA1c 6.4–10%, Israel	150 mL mineral water) per day in the evening for 2 years Mey Eden, different European sources, all low mineralized	CG1: 150 mL red wine CG2: 150 mL white wine	FPG HOMA-IR (all PO) Fasting insulin HbA1c (all SO) HDL-Chol, Apolip-a (PO)	Water group sign. Increase of FPG and HOMA-IR compared to white wine. No sign. difference for HbA1c, fasting insulin and outcomes compared to red wine	ITT, no blinding
Rafraf et al., 2015 [24] & Zemestani et al., 2016 [31]	RCT parallel 2 groups single-blind	64 type 2 diabetes (males and females) aged 30–60 y Iran	150 mL hot water three times per day immediately after meals for 8 weeks.	150 mL chamomile tea (3 g/150 mL hot water)	FPG Fasting Insulin HOMA-IR HbA1c	Chamomile tea group sign. Decrease for HbA1c (*p* = 0.023), serum insulin levels (*p* < 0.001), HOMA-IR (*p* < 0.001), and no sign. difference for FPG compared to hot water	ITT, no drop-outs, single-blind, sign. difference for FPG, insulin and HOMA-IR at baseline, no PO specified

CG: control group; PO: primary outcome; SO: secondary outcome; sign.: *p* < 0.05; RCT: randomized controlled trial; FPG: fasting plasma glucose; GTT: glucose tolerance test; CVRF: cardiovascular risk factors; y: years.

**Table 4:** Randomized controlled trials (RCTs) comparing low mineralized water with bicarbonate-rich water with low magnesium.

Author/y	Study Type	Population	Intervention	Comparison	Outcome	Results	Risk of Bias
Schoppen et al., 2004 [25]	RCT crossover 2 groups	18 healthy women, >1 postmenopausal, BMI < 30 kg/m^2^, Spain	1 L per day for 2 months Bicarbonate-rich carbonated water: HCO^3−^ 2094 mg/L Cl^−^ 583 mg/L Na^+^ 1116 mg/L Mg^2+^ 6 mg/L	Uncarbonated water: HCO^3−^ 71 mg/L Cl^−^ 6 mg/L Na^+^ 9 mg/L Mg^2+^ 3 mg/L	FPG (SO) CVRF (PO)	Bicarbonate rich water sign. (*p* < 0.001) decreased FPG compared to control water (5.54 vs. 5.17 mmol/L)	ITT, 0/18 drop-outs, no blinding, randomization and allocation not reported
Toxqui & Vaquero, 2016 [26]	RCT crossover 2 groups single-blind	64 healthy adults 18–45 y, with moderately elevated cholesterol (5.2–7.8 mmol/L), Spain	1 L of the test water per day for 8 weeks followed by an 8-week washout period Bicarbonate-rich carbonated water: HCO^3−^ 2050 mg/L Cl^−^ 622 mg/L Na^+^ 1090 mg/L Mg^2+^ 5.8 mg/L	Uncarbonated water: HCO^3−^ 75 mg/L Cl^−^ 4.8 mg/L Na^+^ 7.6 mg/L Mg^2+^ 2.8 mg/L	FPG Fasting insulin (all SO) Cholesterol (PO)	No sign. difference for FPG and fasting insulin between groups Sign. reduction of fasting glucose and LDL—cholesterol compared to baseline in both groups.	No ITT, 8/72 drop-outs, no blinding,
Pérez-Granados et al., 2010 [27]	RCT crossover 2 groups single-blind	18 healthy adults 18–40 y, with moderately elevated cholesterol (>5.2 mmol/L), Spain	1 L of the test water per day for 8 weeks followed by an 8-week washout period Bicarbonate-rich carbonated water: HCO^3−^ 2120 mg/L Cl^−^ 597 mg/L Na^+^ 1102 mg/L Mg^2+^ 9.4 mg/L	Carbonated water: HCO^3−^ 104 mg/L Cl^−^ 11 mg/L Na^+^ 8.7 mg/L Mg^2+^ 5.0 mg/L	FPG Fasting insulin (all SO) Cholesterol (PO)	Bicarbonate-rich water decreased FPG not sign. (*p* = 0.056) and no sign. difference for fasting insulin compared to control water.	No ITT, 10/28 drop-outs, single blind, randomization and allocation not reported
Schorr et al., 1996 [28]	RCT crossover 3 groups double-blind	16 healthy adults 60–72 y, salt reduced diet (<2.3 g/day) Germany	1.5 L of the test water per day for 4 weeks with a 2-week washout period before the control water Bicarbonate-rich water: (1.) HCO^3−^ 879 mg/L Cl^−^ 1507 mg/L Na+ 1295 mg/L Mg^2+^ 9.7 mg/L (2.) HCO^3−^ 1983 mg/L Cl^−^ 152 mg/L Na^+^ 602 mg/L Mg^2+^ 53 mg/L	Uncarbonated water: HCO^3−^ < 5 mg/L Cl^−^ < 5 mg/L Na^+^ < 5 mg/L Mg^2+^ < 1 mg/L	FPG Fasting insulin GTT (all PO)	No sign. difference between the groups.	No ITT, 5/21 drop-outs
Zair et al., 2013 [29]	RCT crossover 2 groups double-blind	12 healthy men, 20–60 y, BMI 18.5–25 kg/m^2^, Cholesterol 2.2–3 g/L, France	1.25 L per day for 8 weeks Bicarbonate-rich carbonated water: HCO^3−^ 4168 mg/L Cl^−^ 329 mg/L Na^+^ 1626 mg/L Mg^2+^ 11 mg/L	Carbonated water: HCO^3−^ 183 mg/L Cl^−^ 48 mg/L Na^+^ 31 mg/L Mg^2+^ 12 mg/L	FPG (SO) Blood lipids (PO)	No sign. difference between the groups for FPG.	ITT, 0/12 drop-outs, randomization and allocation not reported, blinding difficult due to taste of water

PO: primary outcome; SO: secondary outcome; sign.: *p* < 0.05; RCT: randomized controlled trial; FPG: fasting plasma glucose; GTT: glucose tolerance test; CVRF: cardiovascular risk factors; y: years.

**Table 5:** Randomized controlled trials (RCTs) comparing low mineralized water with bicarbonate-rich water with magnesium-rich water.

Author/y	Study Type	Population	Intervention	Comparison	Outcome	Results	Risk of Bias
Gutenbrunner, 1993 [16]	RCT parallel 2 groups single-blind	23 healthy men, 19–31 y, Germany	1.4 L per day of the test waters for 28 days Bicarbonate and magnesium-rich carbonated water: HCO^3−^ 2946 mg/L Cl^−^ 128 mg/L Na^+^ 967 mg/L Mg^2+^ 102 mg/L	Uncarbonated water: HCO^3−^ 150 mg/L Na^+^ 19.8 mg/L Mg not mentioned	FPG Fasting insulin GTT Fructosamin (all PO)	Bicarbonate and magnesium-rich water sign. decreased glucose tolerance and fructosamin compared to control water, but not FPG and fasting insulin.	No ITT, 1/24 drop-outs, single blind, randomization and allocation not reported
Murakami et al., 2015 [30]	RCT parallel 2 groups	19 healthy (7 men, 12 women), 47 y (26–59 y), Japan	500 mL per day premeal, mineral or control water for one week in two cycles. The intervention lasted 4 weeks Bicarbonate and magnesium-rich uncarbonated water: HCO^3−^ 2485 mg/L Cl^−^ 182 mg/L Na^+^ 412 mg/L Mg^2+^ 291 mg/L	Uncarbonated water: HCO^3−^ 28 mg/L Cl^−^ 11 mg/L Na^+^ 10 mg/L Mg^2+^ 1.9 mg/L	FPG Glycoalbumin, Fasting insulin HOMA-IR (all PO) Microbiome	Bicarbonate and magnesium-rich water sign. decreased glycoalbumin compared to control water. No sign. differences for FPG and fasting insulin. Lean-inducible bacteria was increased	No ITT, 7/26 drop-outs, no blinding

CG: control group; PO: primary outcome; SO: secondary outcome; sign.: *p* < 0.05; RCT: randomized controlled trial; FPG: fasting plasma glucose; GTT: glucose tolerance test; CVRF: cardiovascular risk factors; y: years.
